# The efficacy and safety of the Timothy grass allergy sublingual immunotherapy tablet in Canadian adults and children

**DOI:** 10.1186/1710-1492-10-53

**Published:** 2014-10-30

**Authors:** Jacques Hébert, Michael Blaiss, Susan Waserman, Harold Kim, Peter Creticos, Jennifer Maloney, Amarjot Kaur, Ziliang Li, Harold Nelson, Hendrik Nolte

**Affiliations:** Centre de Recherche Appliquée en Allergie de Québec, Québec, Canada; University of Tennessee Health Science Center, Memphis, TN USA; McMaster University, Hamilton, ON Canada; Western University, London, ON Canada; Johns Hopkins University School of Medicine, Baltimore, MD USA; Creticos Research Group, Crownsville, MD USA; Merck & Co., Inc, Whitehouse Station, NJ USA; National Jewish Health, Denver, CO USA

**Keywords:** Allergic rhinitis, Conjunctivitis, Timothy grass pollen, Sublingual immunotherapy tablet

## Abstract

**Background:**

The effect of sublingual Timothy grass immunotherapy tablet 2800 BAU (grass SLIT-T) has been evaluated in three North American trials in adults and children who have allergic rhinitis with or without conjunctivitis (AR/C). This paper examines the effects of grass SLIT-T in Canadians.

**Methods:**

Data for grass-allergic Canadians in three randomized, placebo-controlled, double-blind trials were analyzed post hoc: 1) adults ≥18 y, grass-pollen season [GPS] 2009; 2) children 5– <18 y, 2009; and 3) adults 18–65 y and children 5– <18 y, GPS 2012. Data from the GPS 2009 trials were pooled to provide a more precise estimate of treatment effects than the individual studies would provide. In every trial, participants received once-daily grass SLIT-T or placebo approximately 12 weeks before and continuing throughout the GPS. Participants used daily electronic diaries to record AR/C symptoms and medication use for treatment of symptoms. The therapeutic effect of grass SLIT-T was measured as a total combined score (TCS = daily symptom score + daily medication score) averaged over the entire GPS. Safety was assessed by monitoring adverse events (AEs).

**Results:**

In the three trials, 386 Canadian participants were randomized; the overall population had 2284 participants. Canadian participants treated with grass SLIT-T in the pooled adult-pediatric 2009 trials showed a 38% mean TCS reduction relative to placebo (-2.06 difference [95% CI: -3.72, -0.39]; 3.32 vs. 5.37). Participants treated with grass SLIT-T in the adult-pediatric 2012 trial showed a 37% median TCS reduction relative to placebo (-1.53 difference [95% CI: -2.1, -0.3]; 2.58 vs. 4.11). Similar efficacy findings were observed over the peak GPS. Approximately 90% of treatment-related AEs were mild or moderate in severity. Two Canadian participants had moderate systemic allergic reactions (skin, respiratory, abdominal symptoms) to grass SLIT-T; symptoms resolved within 1 hour without medical intervention or treatment. No serious or life-threatening treatment-related AEs occurred.

**Conclusion:**

The 2800 BAU Timothy grass SLIT-T significantly improved AR/C induced by Timothy grass pollen in adults and children ≥5 y in Canadians, which was consistent with the robust efficacy observed in the overall trial population. The treatment was generally well tolerated.

**Trial registration:**

Clinicaltrials.gov identifiers NCT00562159, NCT00550550, NCT01385371.

## Introduction

Upper respiratory allergies, including allergic rhinitis with or without conjunctivitis (AR/C), are estimated to affect 20% to 25% of Canadians [1]. In the International Study of Asthma and Allergies in Childhood, up to 24% of Canadian children in two regions (Saskatoon, SK, and Vancouver, BC) reported in 2003 that they “ever had hay fever” [2]. In 2013, however, the Public Health Agency of Canada reported that nearly 50% of Canadian children have hay fever [3]. AR/C is typically induced by inhalant allergens such as dander, dust mites, and pollens. In North America, grass pollen is one of the most common seasonal allergens that cause allergic symptoms. Many patients with AR/C are allergic to Timothy grass (*Phleum pratense*). Timothy grass pollen is common in all Canadian provinces. In Canada, the prevalence of sensitivity to grass pollen has been found to vary between 14% and 30% in patients with AR/C [4]. Timothy grass is highly cross-reactive with other Northern pasture grasses, such as perennial rye, meadow fescue, bluegrass/june, orchard/cocksfoot, redtop/bent/velvet, and sweet vernal [5–7]; studies of single-grass (ie, Timothy) immunotherapy have demonstrated efficacy against these temperate grasses.

The most common treatment for allergic rhinitis consists of pharmacotherapy. However, as reported in the 2006 Allergies in Canada survey on use of nasal allergy medications, at least two-thirds of participants reported that, even with proper pharmacotherapy, nasal allergic symptoms are associated with lifestyle limitations [8]. Unlike pharmacotherapy, allergen immunotherapy modulates the basic immunologic mechanisms of the allergic disease. Therefore, grass allergen immunotherapy is an important alternative or complementary treatment to pharmacotherapy, with a distinct mechanism of action that induces a sustained long-term effect even after treatment. It is the only treatment known to provide long-term benefit and alter the course of respiratory allergic disease when used in a sustained fashion that is different from what is being presented here [9–12].

One purported advantage of sublingual administration versus subcutaneous immunotherapy administration is improved safety, allowing for self-administration at home after treatment is initiated under physician supervision [12]. Although sublingual immunotherapy tablets (SLIT-T) have been in use in Europe for several years, the experience with Timothy grass immunotherapy tablet in Canadian and US subjects is limited. Given the prevalence of AR in Canada [2] and the relatively small number of AR clinical trials conducted in Canada from 2001 to 2011 [13], it was of interest to determine if SLIT-T would reduce AR symptoms and medication use in Canadian participants. The efficacy and safety of the Timothy grass 2800 BAU SLIT-T, which dissolves within seconds [14], has been investigated in 3 North American trials in which it was found to be effective and well tolerated for participants with grass-pollen–induced AR/C [14–16]. This paper presents results of a post hoc analysis to assess the efficacy of grass SLIT-T for the treatment of grass-pollen–induced AR/C for Canadian participants enrolled in these 3 randomized, placebo-controlled, double-blind trials. The results are from 2 trials conducted during the 2009 grass pollen season (GPS) and 1 trial conducted during the 2012 GPS, which is the largest SLIT-T trial ever conducted [14].

## Methods

### Post hoc analysis design

A post hoc analysis of key efficacy endpoints for the subgroup of Canadian participants was performed for 3 studies of similar design conducted in 2009 or 2012. One 2009 study (P05238; clinicaltrials.gov registration NCT00562159) involved adults (aged 18–65 y) from 9 centers in Canada, and the other 2009 study (P05239; clinicaltrials.gov registration NCT00550550) involved children and adolescents (aged 5–17 years) from 10 centers in Canada. The 2012 study (P08067; clinicaltrials.gov registration NCT01385371) had a population which involved participants of all age groups (aged 5–65 years) from 25 centers in Canada. All participants had symptoms of grass-pollen–induced AR/C with or without asthma. Sensitivity to grass was confirmed by IgE reactivity to Timothy grass extract (average skin prick test wheal diameter ≥5 mm larger than saline control after 15–20 min and serum-specific IgE to *Phleum pretense* ≥0.7 kU/L). A washout period (≥30 days) for inhaled corticosteroids was required before the preseasonal visit (approximately 2 weeks before the start of the GPS) [14]. Study methods have been published/presented for each study and are described elsewhere [14–16].

Data for Canadian participants in the 2 trials conducted in the 2009 GPS were pooled for the present analysis. Pooling of these 2 trials from the same season delivers a more precise estimate of treatment effects than could be provided by the individual 2009 trials, which had small Canadian subpopulations. In addition, pooling of results from the adult and pediatric 2009 trials permits side-by-side examination of these results with those from the adult-pediatric 2012 trial in a uniform population of Canadian children/adolescents and adults (aged 5–65 y) across different pollen seasons, as it is known that grass pollen levels may influence study outcomes [17].

### Design of trials included in the post hoc analysis

The primary hypothesis tested for each of the 3 trials was that administration of Timothy grass 2800 BAU SLIT-T compared with placebo would result in superior improvement in total combined score (TCS; rhinoconjunctivitis daily symptom score [DSS] plus daily medication score [DMS]) averaged over the entire GPS. The studies were conducted in accordance with the Declaration of Helsinki and in compliance with Good Clinical Practice guidelines. The protocol was approved by institutional review boards for each center. Randomized subjects were treated once daily with either grass SLIT-T or placebo for approximately 12 or 16 weeks before the GPS and through the entire GPS, for a total of ≥23 weeks post-randomization.

Grass SLIT-T does not require up-titration, as the first dose is considered the maintenance dose and if tolerated will not require participant dose adjustments subsequently (eg, during season). The first 3 consecutive daily doses of grass SLIT-T were administered at the physician’s office in the pooled adult-pediatric 2009 trials, while only the first dose was administered in the physician’s office in the adult-pediatric 2012 trial due to the overall favorable safety profile shown in previous studies [15, 16]. Following initial supervised tablet administration in the three trials, all subsequent doses were self-administered by participants or their guardians (in the case of children) at home. As an added safety precaution, participants were supplied with self-injectable epinephrine and instructed to have it available for up to 30 minutes after daily dosing; they were also given instructions on how and when to use it. In addition, open-label rescue medications for AR/C and asthma symptoms were provided and allowed to be used during the GPS.

### Grass-pollen season

The GPS was defined as beginning on the first of 3 consecutive days with a grass-pollen count ≥10 grains/m^3^ and ending on the last day of the last occurrence of 3 consecutive days with a grass-pollen count ≥10 grains/m^3^. Peak season was defined as the 15 consecutive days within GPS with the highest 15-day moving average pollen count for each site.

### Clinical efficacy assessments

The primary efficacy endpoint in all 3 trials was the TCS averaged over the entire GPS. Key secondary efficacy endpoints in all 3 trials, which were analyzed only if the primary efficacy analysis was significant, were the entire-season DSS, the entire-season DMS, and the peak-season Rhinoconjunctivitis Quality of Life Questionnaire score (not reported here). The peak-season TCS was a key secondary endpoint in the adult-pediatric 2012 trial and a secondary endpoint in the pooled adult-pediatric 2009 trials. Peak-season DSS and DMS were secondary endpoints in all 3 trials [14–16].

Symptoms and medication use were recorded once daily in the evening before bed in an electronic diary. Safety endpoints included adverse events (AEs) [14–16].

### Statistical analysis

For the 2 pooled adult-pediatric 2009 trials, the primary and secondary efficacy endpoints were evaluated using a linear model with asthma status, study site, and treatment group as fixed effects. The efficacy analysis for all trials was in the full-analysis set population (≥1 dose of study medication and ≥1 efficacy measure during GPS), based on the intent-to-treat principle. Subjects were analyzed according to the treatment arm to which they were randomized. Subjects were included in the entire-season or peak-season analysis if they had at least one post-baseline diary record within the defined pollen season. A two-sided 95% CI of the difference in adjusted means between the two treatment groups was presented. For the 2012 between-treatment comparison, the point estimate of the treatment difference was based on the median and the 95% CI for the median difference was based on the Hodges-Lehmann estimator. Median scores within each group were reported to represent the treatment effect for TCS and DSS; the average DMS over the entire GPS was analyzed using a zero-inflated log-normal model due to excessive 0 values in the data; the model adjusted for treatment, baseline asthma status, age category (<18 years or ≥18 years), and pollen region as covariates. Nominal *P* values are reported for the between-treatment differences. No adjustment in multiplicity is made due to post hoc nature of these analyses.

## Results

### Baseline demographics

The Canadian subpopulation randomized to grass SLIT-T or placebo included 386 participants across three trials. The pooled adult-pediatric 2009 trials had 57 adults and 46 children, and the adult-pediatric 2012 trial had 262 adults and 21 children (Table 1). Participants were randomized in the provinces of Ontario and Quebec in the 2009 trials and the provinces of Ontario, Quebec, and British Columbia in the 2012 trial.

**Table 1:** **Demographics and baseline characteristics of the Canadian subgroup in all three trials**

	2009 studies				2012 study	
	Adults		Children		Adults + children*	
Characteristic	Placebo (n =33)	Grass SLIT- T (n =24)	Placebo (n =23)	Grass SLIT- T (n =23)	Placebo (n =141)	Grass SLIT- T (n =142)
Male, %	45	46	57	61	59	49
Age, mean y	35.6	32.8	12.4	11.0	35.7	33.6
White, %	82	92	91	87	81	85
Asthma, %	36	25	17	17	21	25
Polysensitized, %	88	96	91	74	89	89

*The 2012 study involved 262 adults and 21 children.

### Grass-pollen season

The 2009 GPS in Canada had low mean pollen counts of 19 to 21 grains/m^3^ over the entire season, which lasted a median of 52 to 57 days in North America overall. The 2012 GPS in Canada had mean pollen counts of approximately 23 grains/m^3^ over the entire season which lasted a median of 55 days in North America overall. Both of these grass pollen seasons in North America were characterized by few pollen peaks, with average peak season counts of 40 and 53 grains/m^3^, respectively, in 2009 and 2012.

### Primary efficacy results

**Figure 1:**
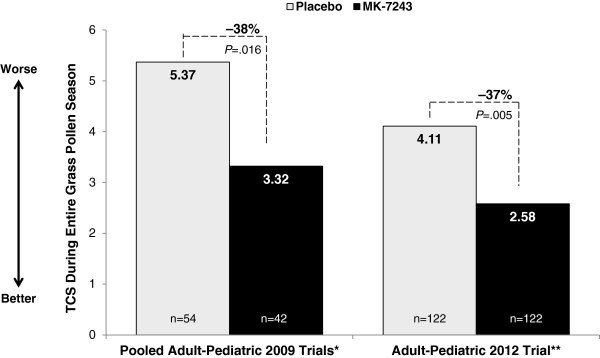
**TCS in Canadian participants over the entire grass pollen season in the pooled adult pediatric 2009 trials and the adult-pediatric 2012 trial.**

Canadian participants taking grass SLIT-T (n =42) in the pooled adult-pediatric 2009 trials showed a 38% mean TCS reduction relative to placebo (n =54), with a -2.06 difference (95% CI: -3.72, -0.39) between grass SLIT-T (3.32) and placebo (5.37; Figure 1). Canadian participants taking grass SLIT-T (n =122) in the adult-pediatric 2012 trial showed a 37% median TCS reduction relative to placebo (n =122), with a -1.53 difference (95% CI: -2.1, -0.3) between grass SLIT-T (2.58) and placebo (4.11; Figure 1).

### Secondary efficacy results

**Figure 2:**
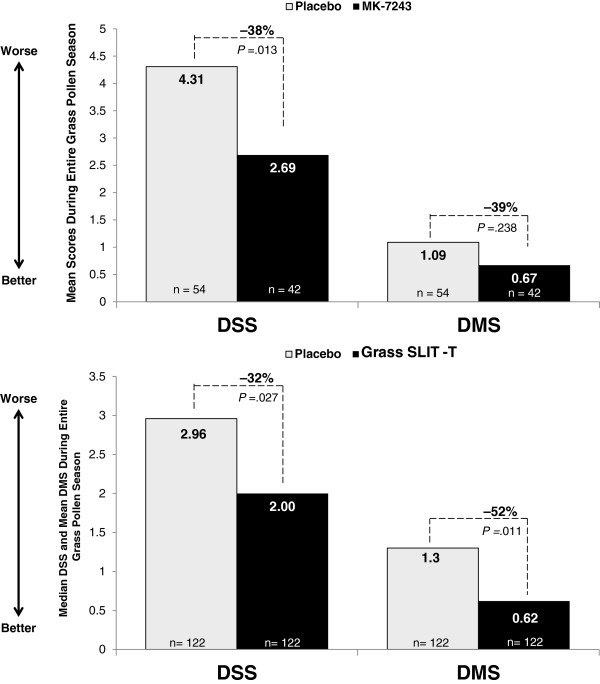
**Symptom and medication scores in Canadian participants over the entire grass pollen season in the pooled adult-pediatric 2009 trials (A) and the adult-pediatric 2012 trial (B).**

In addition to the greater reduction of TCS for grass SLIT-T versus placebo in all 3 trials, the pooled adult-pediatric 2009 trials found that grass SLIT-T reduced the DSS (-38%; p = .013) and DMS (-39%; p = .238) compared with placebo over the entire season in the Canadian subgroup (Figure 2A). Moreover, in the Canadian subgroup of the adult-pediatric 2012 trial, grass SLIT-T reduced the entire-season DSS (-32%; p = .027) and DMS (-52%; p = .011) compared with placebo (Figure 2B).

These patterns of improvement were also seen in the efficacy results over the peak GPS. In the pooled adult-pediatric 2009 trials, peak-season reductions for grass SLIT-T versus placebo in TCS, DSS, and DMS ranged from 40% to 60% in the Canadian subgroup (Table 2). In the adult-pediatric 2012 trial, the TCS, DSS, and DMS peak-season reductions for MK-7243 versus placebo ranged from 17% to 54% in the Canadian subgroup (Table 2). These results for the peak GPS indicate that grass SLIT-T is also effective at reducing AR/C symptoms and the need for rescue medications when allergen exposure is at its highest.

**Table 2:** **TCS**, **DSS**, **and DMS during the entire and peak grass pollen season in the Canadian subgroup in all three trials**

	Pooled adult- pediatric 2009 trials		Adult- pediatric 2012 trial	
	Placebo	Grass SLIT- T	Placebo	Grass SLIT- T
**TCS** **–** **entire season**				
n	54	42	122	122
Mean (SE/SD)*	5.37 (0.68)	3.32 (0.65)	4.96 (4.22)	3.34 (2.93)
Median	3.88	1.46	4.11	2.58
Difference (95% CI)**		-2.06 (-3.72, -0.39)		-1.53 (-2.1, -0.3)
Relative difference*		-38%		-37%
p value		.016		.005
**DSS** **–** **entire season**				
n	54	42	122	122
Mean (SE/SD)*	4.31 (0.50)	2.69 (0.51)	3.48 (2.91)	2.59 (2.16)
Median	3.18	1.38	2.96	2.00
Difference (95% CI)**		-1.63 (-2.89, -0.36)		-0.95 (-1.3, -0.0)
Relative difference		-38%		-32%
p value		.013		.027
**DMS** **–** **entire season**				
n	54	42	122	122
Mean (SE/SD)*	1.09 (0.32)	0.67 (0.23)	1.30 (0.36)	0.62 (0.20)
Median	0.12	0.00	0.65	0.00
Difference (95% CI)**		-0.42 (-1.12, 0.28)		-0.68 (-1.20, -0.16)
Relative difference		-39%		-52%
p value		.238		.011
**TCS** – **Peak season**				
n	52	42	120	119
Mean (SE/SD)*	5.73 (0.78)	3.17 (0.62)	5.50 (4.93)	3.70 (3.40)
Median	3.93	1.47	4.48	3.13
Difference (95% CI)**		-2.56 (-4.32, -0.81)		-1.35 (-2.3, -0.2)
Relative difference		-45%		-30%
p value		.005		.014
**DSS** **–** **peak season**				
n	52	42	120	119
Mean (SE/SD)*	4.64 (0.59)	2.77 (0.51)	3.73 (3.29)	2.85 (2.37)
Median	3.33	1.21	2.93	2.43
Difference (95% CI)**		-1.87 (-3.24, -0.50)		-0.50 (-1.3, 0.1)
Relative difference		-40%		-17%
p value		.008		.109
**DMS** **–** **peak season**				
n	52	42	120	119
Mean (SE/SD)*	1.14 (0.35)	0.46 (0.21)	1.77 (2.61)	0.85 (1.87)
Median	0.00	0.00	0.55	0.00
Difference (95% CI)**		-0.68 (-1.42, 0.05)		-1.13 (-1.94, -0.33)
Relative difference		-60%		-54%
p value		.068		.006

*Adjusted means (SE) for the pooled adult-pediatric 2009 trials; raw means (SD) for the adult-pediatric 2012 trial.

**For the pooled adult-pediatric 2009 trials, analysis of variance model was used with asthma status, treatment group, and site as fixed effects and adjusting for different error variation for each treatment group. For the adult-pediatric 2012 trial, the point estimate of the difference was based on the median; the 95% CI for the median difference was based on the Hodges-Lehmann estimator for TCS and DSS. For DMS in this trial, a zero-inflated log-normal model was used with asthma status, treatment group, age category and pollen region as fixed effects. Absolute and relative treatment differences are estimated based on this model, and confidence intervals are estimated based on the model using the delta method.

*DMS*, daily medication score; *DSS*, daily symptom score; *SD*, standard deviation, *SE*, standard error; *TCS*, total combined score.

### Safety results

Grass SLIT-T was well tolerated in the Canadian subpopulation, with treatment-related AEs (TRAEs) that were generally mild to moderate in severity. No Canadian participant in the pooled adult-pediatric 2009 trials reported a severe AE, and only 3 grass SLIT-T participants and 2 placebo participants in the adult-pediatric 2012 trial reported a severe AE, none of which were treatment-related. Treatment-emergent AEs (TEAEs) were mainly transient local reactions in the mouth, throat, and ear. For Canadians in the pooled 2009 trials, 66% and 45%, respectively, of the grass SLIT-T and placebo groups had any TEAE, whereas 45% and 14%, respectively, of the grass SLIT-T and placebo groups had a TRAE. In the adult-pediatric 2012 trial, 66% of the grass SLIT-T group and 49% of the placebo group had any TEAE, whereas 49% of the grass SLIT-T group and 23% of the placebo group had a TRAE (Table 3).

**Table 3:** **Adverse events reported in the Canadian populations of the pooled adult**-**pediatric 2009 trials and the adult**-**pediatric 2012 trial**

	Pooled adult- pediatric 2009 trials		Adult- pediatric 2012 trial	
	Grass SLIT- T (n =47)	PBO (n =56)	Grass SLIT- T (n =142)	PBO (n =140)
**Any TEAE,** **n** **(%)**	31 (66.0)	25 (44.6)	93 (65.5)	69 (49.3)
**TRAE,** **n** **(%)**	21 (44.7)	8 (14.3)	70 (49.3)	32 (22.9)
**Discontinued due to TRAE,** **n** **(%)**	1 (2.1)	0	7 (4.9)	4 (2.9)
**Local application-** **site TRAE,** **n** **(%)**				
Oral pruritus	7 (14.9)	2 (3.6)	30 (21.1)	6 (4.3)
Ear pruritus	9 (19.1)	2 (3.6)	20 (14.1)	5 (3.6)
Edema mouth	0	0	24 (16.9)	6 (4.3)
Eye pruritus	0	1 (1.8)	4 (2.8)	6 (4.3)
Throat irritation	14 (29.8)	1 (1.8)	47 (33.1)	10 (7.1)
Nasal passage irritation	1 (2.1)	2 (3.6)	6 (4.2)	9 (6.4)
Skin pruritus	2 (4.3)	2 (3.6)	6 (4.2)	2 (1.4)

*AE*, adverse event; *TEAE*, treatment-emergent adverse event; *TRAE*, treatment-related adverse event.

In the Canadian subpopulation across the 3 trials, 9 (4.8%) participants taking grass SLIT-T and 6 (3.0%) participants taking placebo discontinued treatment due to AEs. In the pooled adult-pediatric 2009 trials, one 9-year-old male receiving grass SLIT-T discontinued treatment due to dysphagia, ear pruritus, and throat irritation probably related to treatment, and one participant receiving placebo discontinued due to anxiety that was unlikely related to treatment. In the adult-pediatric 2012 trial, 8 grass SLIT-T participants and 5 placebo participants discontinued due to AEs. In the grass SLIT-T group, AEs leading to discontinuation primarily were local allergic reactions (eg, oral pruritus, ear pruritus, and throat irritation). Two adult participants in the adult-pediatric 2012 trial discontinued treatment for events judged to be systemic allergic reactions. One participant in the adult-pediatric 2012 trial discontinued treatment after experiencing mild edema on the lower lips, redness on mouth corners and chin, epigastric discomfort, and dizziness following the second dose of grass SLIT-T. The second participant developed chest tightness and shortness of breath after grass SLIT-T. Both of these events self-resolved without treatment.

None of these AEs were life-threatening, and there were no cases of anaphylactic shock. No participants in any of the 3 studies had serious TRAEs. None of the Canadian participants used epinephrine for AE relief in the 3 trials, although one adult participant in the pooled adult-pediatric 2009 trials used epinephrine in response to an anxiety attack, which is not an indicated or appropriate use for this medication.

## Discussion

In 3 trials, pooled results of treatment with Timothy grass 2800 BAU SLIT-T, a new formulation and administration of allergen immunotherapy, showed significant improvement of symptoms and a reduction in the need for pharmacotherapy caused by grass pollen in Canadian adults and children aged 5 years and older. The symptom improvement was in the context of participants taking rescue medication for symptoms, and the change with grass SLIT-T was seen on top of the effect obtained with the rescue medication. The primary results in the Canadian subgroup of the pooled adult-pediatric 2009 trials and the adult-pediatric 2012 trial confirm the findings of North American and European trials. Reductions of entire-season TCS in the Canadian subgroup ranged from 38% to 39% in the present post hoc analysis, and TCS reductions ranged from 20% to 26% in the overall North American trials [14–16]. In European trials, which had separate analyses of symptom and medication scores, reductions of DSS and DMS ranged from 16% to 38% compared with placebo [18–21]. The North American trials and Canadian subgroup analyses are limited in that treatment effects over 1 season were evaluated, although sustained efficacy has been demonstrated over a 2-year follow up following 3 years of daily treatment with Timothy grass SLIT-T [10]. Another limitation is the nature of the post hoc analyses and the fact that the sample size is relatively small in the Canadian subgroup; therefore, results should be interpreted in terms of overall trends in treatment effect.

The potential for disease modification with sublingual immunotherapy should be considered when comparing grass SLIT-T with medication to treat symptoms. Immunotherapy has been shown to induce allergen-specific IgG antibodies which block allergen-induced IgE-dependent histamine release from basophils [22], and grass SLIT has been shown to reduce serum eosinophil cationic protein levels during pollen season [23]. In addition, grass SLIT-T has been found to induce marked and sustained increases of *Phleum pratense*-specific IgG4 as well as IgE-blocking factor, immunomodulatory effects leading to possible immune deviation and tolerance development, as observed in a long-term clinical trial [10]. The T-cell response is thought to account for disease modification. In 40 trial participants treated with grass SLIT-T, a short-term increase in cytokine-producing (T_H_2) cell frequency and sIgE levels occurred at Month 1, followed by downregulation of T_H_2 frequency from Month 4 onward through 2 years of therapy [24]. Allergen immunotherapy targets the immune system and the underlying cause of AR/C. An induction period (ie, preseasonal treatment) is required to induce clinically relevant immunologic changes [25], and in general, immunologic changes can be observed in vitro as early as 28 days after treatment initiation. However, clinical study experience indicates that an induction period longer than 28 days is required [26, 27]. A treatment effect may be observed with preseasonal treatment of 8 weeks, which further improves with at least 12 weeks of preseasonal treatment. Reduction of symptom and medication scores increased with longer preseasonal treatment, which is evident in a mixed regression model with the P-value approaching zero that was used to analyze data from three trials [26]. The preseasonal treatment period was approximately eight weeks in one trial [21], approximately 12 weeks in a second trial [18], and 16 to 35 weeks in a third trial [28]. Sustained immunological and clinical post-treatment effect for at least 2 years may require up to 3 years of continuous (year-round) treatment [10].

Grass SLIT-T provides an effective new modality for treating grass pollen–allergic individuals with Timothy (and other cross-reactive *Festucoideae*) grass-pollen–induced AR/C. Once-daily treatment with grass SLIT-T does not require up-titration of the administered dose, which simplifies the treatment regimen. In addition, only the first dose of grass SLIT-T is administered in the physician’s office, whereas subsequent doses are self-administered at home. Overall safety profiles of grass SLIT-T in the studies were similar, and no new safety concerns emerged from the studies. Local application site reactions (eg, oral pruritus) were the most common TRAEs; these events were generally mild to moderate in severity and tended to occur early in treatment. The two participants in the 2012 study who discontinued treatment due to a systemic allergic reaction recovered on their own completely with no need for treatment or additional medication.

Grass SLIT-T significantly improved AR/C caused by grass pollen in Canadian adults and children aged 5 to 65 years. Improvement was shown early in the grass pollen season and was maintained throughout the season, with a similar or greater treatment effect during the peak pollen exposure. The treatment was well tolerated for up to 34 weeks of treatment, and no new safety signals emerged from these trials. In conclusion, the data indicate that grass SLIT-T provides an effective and well-tolerated new immunotherapy modality for treating participants with grass-pollen–induced AR/C.

## Authors’ information

Dr. Blaiss was employed by the University of Tennessee when the studies took place, but is now employed by Merck & Co., Inc., North Wales, PA.
